# “I Know That It's Something That's Creating a Bond”: Fathers' Experiences of Participating in Baby Theater With Their Infants in South Africa

**DOI:** 10.3389/fpsyt.2020.580038

**Published:** 2020-11-11

**Authors:** Brenda Cowley, Anusha Lachman, Elvin Williams, Astrid Berg

**Affiliations:** ^1^Department of Psychiatry, University of Stellenbosch, Cape Town, South Africa; ^2^Department of Occupational Therapy, University of Stellenbosch, Cape Town, South Africa; ^3^Division of Child and Adolescent Psychiatry, University of Cape Town, Cape Town, South Africa

**Keywords:** father-infant interaction, involvement, father's experience, South Africa, Baby Theater

## Abstract

In many African countries, particularly those with largely patriarchal societies like South Africa, promoting father-child interaction can pose a challenge. An informative medium that could potentially encourage active participation in pleasurable interactions between fathers and babies may prove to be an important way in which to stimulate fathers' awareness of their infants' abilities. A Cape Town based theater company created the first ever South African baby play for care-givers and their babies between the ages of 2 weeks to 12 months. The play is performed in a contained, relaxing space and offers carers and babies time to relate to each other in a pleasurable atmosphere, while specially trained actors model sensitive and responsive interactions. Baby Theater could be a way to encourage fathers' involvement with their infants, however, no research is currently available documenting fathers' perceptions about Baby Theater experience.

**Aim:** To explore fathers' experience of participating in Baby Theater.

**Method:** This qualitative study involved six fathers who, with their infants, participated in the Baby Theater production. A week later the fathers were divided into two focus groups to give them the opportunity to discuss their thoughts about the experience and to reflect on whether it had any subsequent impact on their interactions with their babies. The audiotaped, transcribed material was thematically analyzed using an interpretative phenomenological approach.

**Results:** The fathers described the experience as educative and enjoyable. They reported that the program had a positive impact on the way they interacted with their infants and also positively influenced their relationship with them. Additionally, they reported feeling more confident about coping with their babies on their own, and appreciated the connection with the other fathers in the group. Cultural, societal, and gender issues were also considered.

**Conclusions:** The subjective experience of the fathers was positive. Further research is needed to assess the lasting effects of the Baby Theater experience.

## Introduction

The importance of early mother-infant attachment and bonding in the promotion of healthy child development has been well-established (1–4). In contrast, relatively few studies have addressed the father-infant relationship despite awareness of the father's unique contribution to the development of his infant (5). Ramchandani et al. (6) found that fathers who were disengaged and remote in their interactions with their 3 month old infants predicted externalizing behavioral problems in the baby at 1 year of age. A positive father-child relationship is associated with better self-regulation and lower levels of childhood aggression in boys and higher self-esteem in girls (7). In addition, positive father involvement results in children showing better cognitive outcomes in later life (8). There is also strong evidence that a father's involvement in the early lives of his children positively impacts their physical and social-emotional development (5, 8, 9).

In many countries, particularly those with largely patriarchal societies like South Africa (10–13), promoting father-child interaction can pose a challenge (14).

Representations in the media and research literature emphasize the increasing number of absent fathers in South Africa (15). Recently, the Stats SA General Household Survey (16) revealed that as many as 43.1% of children lived only with their mothers. While a large number of fathers refuse to acknowledge their paternity and have no contact with their children (17), others are forced to leave their families to find work elsewhere (18). Richter et al. (19) describe this trend of absent fathers as an “epidemic,” especially amongst young fathers. However, Morrell (14), notes that the quality of fatherhood cannot be measured simplistically in relation to his physical presence or absence from the household. That is, fathers who are physically absent may nevertheless be emotionally present in the lives of their children and, conversely, physically present fathers can be emotionally absent.

South Africa is a patriarchal country in which the role of the father as the primary authority figure in the household has a powerful impact on family life (11). Patriarchy in South Africa today is considered by many to be a “serious societal illness, and often the root contributor of violence against women and children” (17, p. 304).

The roots of South African patriarchy are complex and are primarily embedded in traditional African Culture (10), a colonial past (20), and the deleterious effects of apartheid (13). It has woven complex gender dynamics into the fabric of South African society which are entrenched into both the Eurocentric and Afrocentric cultures within the country (21).

In 1998 the Commission on Gender equality stated that (22):

“It is a sad fact that one of the few profoundly non-racial institutions in South Africa is patriarchy… indeed, it is so firmly rooted that it is given a cultural halo and identified with customs and personalities of different communities” (p. 10).

Little has changed over the years, despite state legislation promoting gender equality (23) and men remain dominate not only in homes but in many other areas including public life, politics, and earnings (24). Most women still traditionally fulfill the role of caregiver, homemaker, and domestic caretaker.

There are some sectors of South African society which hold much more egalitarian views about the roles of men and women within the home and in the wider society. These views have arisen as, progressively, more women have become economically active and independent. This has resulted in a considerably more balanced division of labor between males and females in some homes although South African men generally do less childcare work than women (25). But, as Olivier et al. (13) note, “the change from traditional to egalitarian views does not develop to the same extent everywhere, with larger differences between the roles of men and women in poorer, less developed societies” (p. 12).

There is a general need to establish early South African intervention father-infant programs (26). Since 1990's (the post-apartheid era) a few South African programs have been implemented. These include didactic programs run in clinics and hospitals involving practical instructions on health care and parenting, rather than sensitive or nurturing care for children. Many of these offerings do not include a culturally sensitive or Afrocentric approach to parenting. The existing programs have not been vigorously assessed and the benefits of these kinds of didactic educational programs remains unclear (27).

Some programs are run by civil society organizations such as the South African Men's Forum Fatherhood Project and The Sonke Gender Justice Programme. In 2012, the Sonke Gender Justice Programme introduced a “MenCare” initiative which is a global multidimensional program focusing on boys and men between the ages of 15 and 35. Their aim is “to create a society where men are engaged as caregivers and fathers, and where gender equality is a reality in the family context” (12, p. 17). Their programs include 12 week long community based father groups in which men are encouraged to share their experiences of fatherhood, are taught practical parenting and communication skills and are encouraged to provide maternal support. The MenCare+ South African Outcome Measurement Report (13) showed a significant change in pre- and post-intervention scores in a number of areas which included a significant change in the men's attitudes relating to gender norms. In the parenting groups, the fathers also showed an increase in their gender equitable attitudes. In addition, the report states that the percentage of men attending prenatal care visits increased after the program (13).

A systematic literature review by Magill-Evans et al. (28) concludes that the most effective interventions with fathers of infants and toddlers are those which involve the father actively participating with or observing his own child. A useful way of engaging fathers in this kind of activity may be involving them in performances such as theater or puppet productions. An example of this would be the Theater for Early Years (TEY) which has existed for at least four decades, having started in London with a pre-school production called “Exploding Punch and Judy” in 1981.

There has, however, been controversy over the usefulness of Theater for the Early Years, partly because the early performances were not tailored to the needs of very young children who were regarded as a difficult audience (29). But more recent developmental research and an understanding of neuroscience has changed the nature of performances (29). In recent years the scope of TEY has widened to include young audiences with autism and other cognitive disabilities (30).

Infants' sophisticated capacities to absorb and integrate the world through their senses have legitimized this new audience for the performing arts. In 2002, the Oily Cart Theater Company in London produced a show for an audience of very young children ranging in age from 6 months to 2 years. Since then other theater companies around the world have produced Baby Theater performances geared toward children from as young as 2 weeks up to ~2 years of age. These countries include New Zealand (31) Canada (32), the United States, Australia and a number of European countries (33). Each theater constructs its own unique performance and choreography.

With this in mind, we wondered whether a local theater performance specifically designed for babies and their carers might be beneficial in the local context. Our aim was to explore the lived experiences of fathers who participated in a Baby Theater project in South Africa.

Such a theater exists as the Magnet Theatre in Observatory in Cape Town, which is a physical theater company with a focus on community involvement. As a community theater, dedicated to training actors from local communities, it relies heavily on sponsorship and donations. The aim of the donors is to support the development of community arts in South Africa. The theater charges a nominal fee for performances. It has operated for the past 32 years in South Africa and internationally. It is hoping to extend their reach into other venues such as mother-baby clinics and hospitals. It advertises its performances on its website and in the local press. With sponsorship in 2015, the Magnet Theatre was able to host a Northern Ireland based company, Replay Theater, which specializes in theater for the very young. An example of Replay Theater's work, ***TiNY***, for the under 1 year olds, was presented in a number of performances to parent-infant audiences in Cape Town. These were well-received and allowed Magnet's Early Years Theater Company members to observe the impact of the production and provided insights into the contexts in which a South African production, of the same nature, could operate.

Inspired by Replay Theater's incubation, the Magnet Early Years Theater company created ***SCOOP: Kitchen play for Carers and Babes***: The first ever South African baby play for care-givers and babies between the ages of 2 weeks−12 months. It is performed in a contained and relaxing space of a tailor-made tent and accommodates six carers and babies at a time. ***SCOOP*** created a unique opportunity for carers to explore the possibilities of meaningful engagement with their babies. At the heart of this project was a drive to create greater awareness of the crucial importance of strong emotional attachments in infants' lives and the direct effect it has on the development of their brains. It is available for anyone who is a caregiver of a baby and for them to be able to walk away with a sense of how responsive and attentive their babies are, even from the earliest age. It is hoped that they might possibly continue to engage, connect, sing, and play with their little ones, contributing to secure attachment.

The authors of this study were looking for a novel way of encouraging fathers to interact with their babies. They approached the Magnet Early Years Theater Company who agreed to put on their performance for the father-infant study. This would allow fathers and their babies take part and, at the same time, would be a fresh way of enhancing the father's awareness of his infant's abilities. This would be a way to stimulate his curiosity in what his infant may be thinking and feeling, thereby increasing paternal involvement.

The aim of this study was to explore the lived experience of fathers' participation in Baby Theater and the potential impact that this would have on their relationship with their infants. The infants who participated in the study were between the ages of 4–11 months.

As the period between birth and 12 months of age makes use of mostly non-lexical language and is focussed on sensory stimulation, it was felt that the Baby Theater would be meaningful across the cultural spectrum. After 3 months infants have a sense of their “core self” and are able to be with another person with whom they are able to interact (34). After 12 months a new developmental phase sets in and language starts to appear and become progressively prominent. Our interest was in how fathers related to their infants during the earlier, pre-verbal stage of development.

## Methods

### Study design

A phenomenological research design was chosen as a framework for this research because it provides rich data by offering a detailed examination of the “lived experience” of the participants instead of working with “pre-existing theoretical preconceptions” [(35), p. 41]. An interpretative phenomenological analysis was used because of the idiographic nature of this research (36) and the small sample size. Focus groups were conducted with the six fathers who had participated in the Baby Theater production.

### Participants

Purposive sampling was used to ensure homogeneity of the research participants.

The Magnet Theatre has an established Baby Theater program and therefore they had a database of potential dyads from which to recruit. An electronic invitation was sent to all the addresses on their database. In addition, flyers advertising the study were handed to a number of passers-by in the street outside the theater. Flyers were also distributed to a number of church groups.

Almost all the caregivers who attend the Magnet Theatre Scoop Performance are mothers and female child carers. They come to the performance hoping to share an enjoyable time with their infants. For the most part, few fathers attend the Scoop performances. Fathers who attend with a second parent or grandparent are not primarily “responsible” for the experience of the baby in the theater, and therefore slightly less engaged. The fathers in our study were similar to the wider group of fathers attending Baby Theater in terms of age, language, and race. They were part of the first show involving father-infant dyads only. Having the babies under their sole care during the performance, meant that they needed to be more engaged with their babies.

Eligible participants were biological fathers who had contact with their young babies but who were not necessarily living in the same household as their infant. The inclusion criterion for the babies was that they had to be below the age of 12 months. Before this age, infants are pre-verbal, relying on sensory stimulation and non-lexical language. This is often the most challenging time for parents who may think that their infant lacks the abilities to interact. The babies who took part in the study were between the ages of 2–11 months. By having a group of babies who were all in this pre-verbal stage of development allowed us to obtain a rich description of the fathers' perceptions during and after the baby theater experience.

Initially, the Magnet Theatre recruited 14 fathers who were invited to participate in different arms of the study, including a quantitative one which aimed to assess the quality of the interactions of a select sample of South African fathers using the Coding Interactive Behavior Scale (37). Additionally, the aim was to determine whether the intervention of Baby Theater could improve the quality of father-infant interactions. The results of this part of the study were inconclusive because the sample was too small. Two of the fathers dropped out of the study. One was excluded because he did not return for the second CIB assessment and another had a baby who was too young to meet the CIB criteria.

For the purposes of our study, focus groups were established to assess the qualitative responses of the fathers. Six fathers who had been randomly chosen to be in the experimental group of the CIB study took part in the focus groups. Three of the fathers were black African, one was white and two were of colored descent^1^. They lived in areas which traditionally accommodated families from mid to low socio-economic backgrounds.

### Procedures

The fathers made voluntary contact with the theater indicating their willingness to partake in the study. Inclusion and exclusion criteria were applied and six father-infant dyads were included in the study.

They independently completed a consent form in their own language (English, Afrikaans, or isiXhosa) and a demographic questionnaire (Table 1) which asked about ethnicity, marital status, father's age, infant's age, co-habitation with their infants, employment status, and financial provision.

**Table 1:** Brief demographic profile.

**Marital Status**		**Total no. of children**	
Married	3	One	3
Unmarried	3	Two	3
**Fathers' Age range**		**Employed**	
25–29	2	Yes	5
30–35	2	No	1
36–40	1		
**Babies' Age range**		**Financial Provision**	
2–5 months	1	Yes	5
5–9 months	3	When able	1
10–12 months	2		
**Co-habitation**			
Yes	4		
No	2		

Spare nappies (diapers) and wipes were provided for the babies and refreshments were also laid on for the fathers and babies.

The performance was held within the enclosed space of a specially constructed tent with soft, padded flooring. This tent was designed to accommodate four actors and six parent-infant dyads seated on the floor. The play lasted half an hour with an additional 10 minutes for the babies to play freely with the props used by the actors during the performance. Some of the family members including older siblings came to view the show from outside the tent where chairs were placed to accommodate them. This is a customary procedure of the theater.

The play was carefully choreographed in such a way that one activity moved smoothly into another and each was just long enough to attract and hold the babies' attention. The four actors situated themselves at each corner of the tent. They performed the same choreographed actions whilst communicating non-verbally (via facial expression and gesture) with the babies, building up an individual rapport with each baby. A cameraman was present to video some of the interactions of the babies with their caretakers and the actors. These clips were used, not only to document the theater choreography, but were also later threaded into a documentary on the show (see for documentary video^2^). The reasons for the videotaping were explained to the fathers who gave written permission to have themselves and their babies filmed, and the clips added into the documentary. This documentary video was later sent to each father.

For such a performance to be meaningful, it has to be tailored to the infant's needs and capabilities. The approach therefore has to be flexible and sensory over-stimulation has to be avoided. For this, the performers require knowledge about young children's developmental milestones as well as a particular sensitivity that would enable them to read non-verbal cues.

The play began with the babies sitting on their father's laps. As the performance continued, those babies who were able to crawl could move closer to the actors if they chose to do so. The fathers sat on the floor close by, providing a secure base to which the babies could return if needed. Using songs, sounds, lights and textures, the actors took the babies on “an enchanting journey crafted to delight, surprise and soothe them” [(38), p. 11]. Through their interaction with the babies, the four performers revealed innovative ways to connect and communicate with infants. Within the relaxed atmosphere, the fathers were able to observe how the actors interacted with the babies, the babies' enjoyment of the performance, and the infants' individual responses to the various stimuli presented.

### Focus Groups

A week after being involved in the Baby Theater production, the fathers were divided into two focus groups comprising three fathers in each.

Because of space constraints, the theater venue was unable to accommodate the focus groups. These group meetings were therefore held in a small cottage which houses part of the Cape Town University's Division of Child and Adolescent Psychiatry (with which one of the authors is connected). This particular venue allowed the relaxed and informal atmosphere created by the theater performance to continue into the focus group setting.

Having two groups gave the fathers more opportunity to speak within the time available. The structure and approach to the focus group allowed the representation of multiple perspectives, bound by common experience of participating in the baby theater production. The content of the focus group also acknowledged consensus or discrepancies between the fathers' experiences, while providing a reflective space for the fathers' to ascribe meaning to their lived experiences of being engaged in the baby theater production. Each focus group session was an hour long.

The group discussions were audiotaped and later transcribed. The transcription was then analyzed using an interpretative phenomenological approach.

An external facilitator was used to strengthen the trustworthiness of our research. This facilitator is an experienced clinical social worker who had no connection to, or knowledge of Baby Theater. In addition, she had little in depth knowledge of the study, but had a good understanding of young babies. At the time she was a MPhil masters student in Infant Mental Health at the University of Stellenbosch.

Both groups were led by the same facilitator who had had no contact with the fathers before the focus group session and who introduced herself as an outsider to the study. She used semi-structured interviews to guide the discussion. The facilitator was given six open ended questions to use (see below), but instructed to also allow the participants to direct the conversation.

The researcher and supervisor each compiled a set of questions and then met to discuss which of the questions were the most suitable to be included in the semi-structured interviews. The questions were broad and open, allowing for unexpected findings and allowing for the interviewer to probe the responses more deeply.

The focus group questions were the following:

**Focus Group Questions**

1. What did you (the fathers) expect from the Baby Theater?
2. How did you find the experience/feel about the performance?
3. What made it special or what was missing?
4. What do you think your baby experienced?
5. Did you learn something new about your baby? What?
6. Do you think this is a good activity for parents to do with their baby?

### Data Analysis

The transcribed data was thematically analyzed according to the steps outlined by Smith and Osborn (35). The text was read a number of times to familiarize the principle researcher with the material and allow her to start gathering common trends and insights in the comments of the respondents. On further reading, initial notes were transformed into themes. The emergent themes were listed and connections between themes were made. Superordinate and subordinate themes were then isolated. These were checked against the actual words of the participant. Identifiers (references to quotes in the transcript) were given as keywords with page numbers referring back to the original source. Triangulation was used to increase the credibility and validity of the results through researcher-supervisor debriefing. Data saturation was discussed.

In order to further ensure trustworthiness, a process of self-reflection about personal biases and preconceptions is of the utmost importance. All the authors, as well as the facilitator of the focus groups, are from social backgrounds in which more egalitarian views about gender roles are seen as important. This viewpoint, together with a background knowledge of the benefits of a father's involvement in the development of his infant underlay the study. At the same time, the authors expressed a subjective awareness that, despite the largely negative stereotype of South African fathers not being involved with their young children, there are many exceptions. The fact that the fathers reported a positive response to their theater experience, and it seemed to have had an impact on the way that they interacted with their babies at home, was met by the authors with a great sense of pleasure. We are aware that our underlying feelings toward this study, although not directly conveyed to the fathers could have had an influence on the father's responses as will be discussed in the Limitations Section.

### Ethical Considerations

The study was approved by the Health Research Ethics Committee (HREC) of Stellenbosch University (Reference #: S17/10/266). It was conducted in accordance with the South African Good Clinical Practice Guidelines, as well as the Declaration of Helsinki (2013). Participation was voluntary, and all fathers provided written informed consent. As the children were under 12 months, consent was gained from the parent. Participants were compensated as per national HREC recommendations for their time and travel expenses.

## Results

The interpretative phenomenological analysis of the focus group discussions isolated three superordinate and nine subordinate themes, detailed in the Table 2.

**Table 2:** Superordinate and subordinate themes.

**Superordinate Themes**	**Subordinate Themes**
The educative value of Baby Theater's	The simplicity of interaction
	Peer connection
The father-infant dyad	The dyadic connection
	The awareness of the baby's abilities
	The bonding experience
The role of the father	Cultural influences
	Societal Influences
	Gender bias
	Father empowerment

### Theme 1: The Educative Value of Baby Theater

The value of Baby Theater lies in the provision of a learning experience in a novel and enjoyable way.

#### The Simplicity of Interaction

One of the fathers summarized this experience in the following comment:

“*I came there knowing nothing about my baby, but now I come knowing how to interact with my baby*.” YG (cohabiting with baby).

It became clear that most of the fathers felt that they lacked the interactive skills when it came to relating to their babies and they reacted with pleasure and surprise at how simple this can be, for example, using everyday objects such as keys, spoons, plastic cups and bowls to entertain their babies. This came as a new and interesting discovery, as one father remarked:

“*You don't really need all those toys. It taught us that you can be creative with what you have. I think sometimes we forget that*.” LZ (living apart from baby).

An integral part of the baby theater performance was the singing. For example, each of the babies' names were woven into song and various sound effects were also created through voice. This demonstrated that singing can also be used as a positive interactive tool. Until then, some of the fathers had not thought of using their voices for the entertainment of their babies. In addition, one father also realized that the use of his voice could have an impact on a deeper level:

“*I also learnt it's not to make her stop crying, but to communicate and build a friendship*.” JA (cohabiting with baby).

Here, it can be seen that the idea of singing to his child changed from regarding it simply as a way of soothing to a way of connecting on a deeper level—that of “building a friendship” with his infant. This indicates that the value of the Baby Theater experience can extend much further than a demonstration of simple ways to engage babies.

#### Peer Connection

An additional value of Baby Theater in bringing fathers and their infants together as a group is that this appeared to facilitate a connection between the fathers. By watching each other deal with their babies and, through sharing their experiences, there was a sense of a common ground between them.

“…*It's not just me that is not used to this whole father thing and being alone with them and stuff, but it was very nice to see how other dads react, what they also are doing, so you don't feel like you're the only one out there that feels this way and don't know what to do, maybe. It inspired a lot of confidence, also*.” LZ (living apart from his baby).

For another father, being able to watch how the other fathers dealt with their babies was a direct learning experience:

“*The bond between me and my baby is not strong. I need to prepare more time for me and my baby so when I take him maybe for a walk or something, he doesn't cry there and I can't stop him. I learnt a lot from you guys – how you treat your babies and I think that this is going to help me in the future*.” CB (living apart from baby).

### Theme 2: The Father-Infant Dyad

By their very nature, relationships cannot be one-sided and as a result of participating in the theater performance, it would seem that the fathers began to realize this.

#### The Dyadic Connection

In his comment below, a father voiced his awareness that the baby “expects” something from him. His awareness of the baby's expectation indicated that he understood that the baby also plays and active role in the relationship.

“*What I learnt from the theater time—I must play with the child, physically playing. I crawl around, I run and talk. When I get home, I'm not just me, I must know that there is that little one who expects something from me…*” YG (cohabiting with baby).

#### The Increased Awareness of the Baby's Abilities

By having the opportunity to concentrate on his own baby's reactions to the various sensory experiences offered, each father was able to observe the uniqueness of his infant's response. In this way they became aware of how responsive and attentive their babies could be. It was hoped that this realization would inspire the fathers to want to have more contact with their infants.

This father described his experience in the following way:

“… *Every time there was a new sound, you could see her focus. It was almost like she tried to hone in on what was happening. It was interesting to me because sometimes you think that your baby, they don't experience all of that stuff but they actually do*.” JA (cohabiting with baby).

The closeness with which this father observed his baby is evident in his description of the baby's focus: “It was almost like she tried to hone in on what was happening.” By carefully observing his infant it would seem that he was becoming aware that his baby had individual and unique preferences and perhaps, in his mind, his infant was becoming more of a “person.” This perception of the infant “as a person” was implied in similar comments made by a number of fathers. For example, one father stated:

“*It seems like his own personality can be quite focused*.” WG (cohabiting with baby).

The realization that their infants are experiential and responsive individuals inspired a desire in some of the fathers to interact with their babies more. One father stated: “*I need to expose her to much more things to really cultivate her different skills and stuff*.” (TD, cohabiting). Another remarked that “*I can see I could actually do more with him. It creates a bigger bond between us*.” YG (cohabiting with baby).

#### The Increased Bonding Experience

Some of the fathers felt that their relationship with their infants had changed as a result of their participation: “*I can conclude that this program got me closer to my child*.” TD (cohabiting with baby).

Another father spoke about the experience as facilitating the bridging of a gap:

“…* and now every night I can't wait to get home, because I know I'll spend time with her. I am actually disappointed when I go home and she is sleeping. Because the gap was bridged, and that was good for me*.” JA (cohabiting with baby).

### Theme 3: The Role of the Father

The theme of the role of the father is complex in that it touches on cultural, social as well as gender issues.

#### Cultural Influences

South African society is largely patriarchal with traditionally-divided roles. For some fathers, their involvement with their babies in the theater performance provided a new experience from a collective point of view.

One father outlined how, in his community, family roles have been passed down through the generations.

“*Even when we were growing up … we are playing cards with our mother, but our father, he'll be there reading the newspaper or watching the news on the TV, you understand. So that thing is like in your mind: When I have my own family, that is how I must be, you understand. Because if you don't look up to your father, you understand, this is how I must be. So, when you are exposed to this kind of thing—see the good of it*.” TD (cohabiting with baby).

In the above comment, it is clear that it is the mother who is the parental figure who engages with the children, whilst the father is a more distant figure whose focus is on the outside world (reading the newspaper and watching the news). Although distant, he is respected and admired and a figure who one should aspire to emulate. However, the comment of this particular father indicates that he was willing to embrace a different stance.

#### Social Influences

Societal pressures and influences also had a part to play in the lives of some of our fathers. Referencing his own father, one of our fathers stated:

“*He provided for us … but that soft side, it was like he did not want to show his soft side*.” YG (cohabiting with baby).

Another father spoke about getting down to play with the children as “not our thing”.

“*I don't know about you, but as a black man, our role is just to be a father and a husband. To play with the kids and go down … it's not our thing. But now I'm learning to … you understand, I'm not going to worry how is my wife going to look at me and all that*.” *TD (cohabiting with baby)*.

Although not mentioned directly, it would appear that “play(ing) with the kids and go(ing) down” could be viewed in some societies as non- masculine. This can be seen in this father's perception of his wife's possible negative view of him if he adopts a less distant role with his children. As it turns out however, his wife embraced his new way of being with his infant.

“*I know it's something that's creating a bond, you understand. And it's also making my wife happy to see us like that*.” TD (cohabiting with baby).

This suggests that societal norms can be changed, especially within the family context.

#### Gender Bias

A number of fathers mentioned that the Baby Theater experience also had an impact on their view of gender roles within the home:

“…* you can't look only to the (one) side when it comes to the mother and the father. It had just become broad now. Like washing—midweek she was doing our daughter's washing. I was there. I was the one hanging, you know and doing with the StaySoft. so, you share the responsibility. So it has really opened our minds*.” JA (cohabiting with baby).

For this father, it would seem that the connection that he made with his baby during the theater performance had a direct impact on his willingness to take part in everyday baby chores. It would seem that being “there” was not simply in the physical side of hanging the clothes, but in the desire to have an equal share in the responsibility of bringing up his daughter.

This same desire to share equal responsibility was evident in the comment of another father:

“*Me changing a nappy? … I don't know, but I'm okay with it, because (now) even before I come to the nappy changing, I know that she's my responsibility, you understand, not just my wife's responsibility. She's also mine… I know I've got to take the initiative because I took the liberty of being with her*.” TD (cohabiting with baby).

It is clear that the confidence which this father gained through the experience enabled him to feel a joint ownership of his baby (“she is also mine”) which increased his sense of responsibility toward her. In addition, it appears that he is also claiming her in a deeper sense: Having “taken the liberty of being with her” he has discovered a powerful loving connection between himself and his baby daughter. It is this connection which spurs him into wanting to take part in her everyday baby care.

#### Father Disempowerment

Linked to the issue of gender is that of father disempowerment. The role of the mother as the designated baby-carer appeared to have rendered some of the fathers less confident in the day to day care of their infants. As one father described:

“*I'll say a lot of daddies—maybe I was also included—the baby will cry, you pick up the baby while the mother is busy and the baby is crying. You pick up the baby and you* “*shh-shh*” *the baby to stop the crying and the baby is crying and right there then you get* “*gatvol*^3^”. *You just quickly want to pass the child to the mother*” YG (cohabiting with baby).

In this extract, the father related how he usually defers to the “higher expertise” of the mother with some relief. However, he spoke about his different approach after the Baby Theater performance:

“*But now knowing that you can make the baby keep quiet while he looks at you and what you are doing—maybe acting or dancing and singing, you know, you've got that potential in you, you know. Rather than just giving the baby away*.” YG (cohabiting with baby).

It is possible that by “just giving the baby away” the fathers may lose something in the relationship with their infants. It is posited that the process of grappling to sooth the infant could add something to the relationship and, if it is met with success, it is likely to move the father deeper into the relationship with his baby.

For one father, the experience of partaking in the baby theater performance without the mother standing as a buffer between the baby and himself had a powerful effect:

“*I took the liberty of being with her (and) I know now I can be with her, you understand. It was really powerful*.” TD (cohabiting with baby).

Not having the mother around as a “default caretaker” was a pleasurable experience for one of the fathers who spoke about the fun of interacting with his infant “*not waiting for mommy to be around to help out—for this, for that—and actually just take on the responsibility and see how it is for us*.” LZ (living apart from baby).

## Discussion

The comments of the fathers who took part in the study demonstrate that their subjective experience of Baby Theater was overwhelmingly positive, which suggests that Baby Theater participation would be an acceptable way to encourage fathers to become more involved with their babies. All the fathers experienced the theater to be a pleasurable, informative and valuable intervention which enhanced their relationship with their infants.

The authors felt that there were specific aspects of the Baby Theater experience that the fathers, as a group, found particularly beneficial. It seemed that the most important aspect was a feeling of being emotionally closer to their infants. In addition, they appeared to have gained a new sense of confidence when engaging with their babies. We attributed this new sense of confidence to the fact that the actors demonstrated that interacting with babies does not have to be complicated. In addition, the experience of having their babies under their sole care during the theater performance (without the relying on the mother to take over if the baby becomes distressed or niggly) increased this sense of confidence in their ability to deal with their infants on their own.

Given the negative representations of absent fathers in the South African media, and the growing trend of absent fathers recorded in the research literature (19), the current study provides a more positive outlook. Those fathers in our sample who lived away from their infants were nevertheless involved with their babies on a regular basis. All the fathers provided financial support for their infants including the unemployed father who provided financial support whenever he was able to do so.

Contrary to more formalized didactic parenting programs, which are often conducted via medical and social services (39), the Baby Theater performance offered the fathers the opportunity to be in close proximity with their infants and watch them respond pleasurably to the stimulation provided by the specially trained actors. The Baby Theater performance offered the fathers and their babies a novel, pleasurable, and simple educative experience which included the simplicity of relating to babies, as demonstrated by the actors (for example by catching and holding the babies' attention with their voices). This appeared to enable the fathers to grasp the idea that relating to babies does not have to be complicated. By having the opportunity to watch their own infant's responses to the different stimuli provided, perhaps made a greater impression on the fathers than if they had simply been given verbal instructions about how to interact with their children. This is in keeping with the findings of Magill-Evans et al. (28) which indicate that the most effective interventions involve “guided observation of one's own infant with modeling” (p. 263). Our study also showed that it had the added benefit of connecting the fathers with each other.

Clinical and neuroscience research over the past two decades has shown that infants come into this world pre-wired to seek sensory stimulation, with distinct preferences as to the sensations they seek. For many parents, this is new information as, traditionally, babies are considered “too young” to have such nuanced awareness (34). By observing their own baby's unique reactions and preferences, the fathers appeared to have developed a new awareness in this regard. It is possible that it was this new awareness that drew them to feel closer to their infants and sparked their interest in having more interaction with them.

Richter (40) notes that “there are cultural, social and individual differences in how fatherhood is defined and expressed” (p. 55). For a number of the fathers cultural, societal, and gender issues played a role in how they viewed fatherhood. In most traditional African and other families, the role of the father is that of the authority figure, moral overseer, and material provider (19) and this role does not include baby care (41). For some of the fathers, the idea of being a father who gets down and plays with his children and shares responsibility for every day baby care was foreign. However, the experience of Baby Theater may have introduced the possibility that direct involvement with his baby could be enriching both for himself and his infant.

Given the fact that there is now a growing recognition that gender roles are socially determined constructs and therefore flexible (42), it is hoped that the pleasure derived from a closer connection with the baby could go some way toward changing the adherence to the societal definitions of parental roles.

Despite the increasing awareness of how important fathers are in the development of their children, there remains a gender bias against fathers which is still firmly entrenched even in the “mother-centric theories of child development” [(43), p. xiii]. In Western industrialized societies, and, in particular, within middle class socio-economic families we are seeing increasing paternal involvement (44). However, for many fathers in South Africa, this is not the case, and the mother is generally seen as the primary expert and authority in the area of child care (45). This could lead to a feeling of disempowerment in the father in the area of child care resulting in a limitation of his interaction with his baby. It would seem that the participation in the experience of Baby Theater, without the involvement of the mothers, gave our fathers a sense of confidence in their interactions with their infants. This was further supported by the opportunity they had to observe how the other fathers were dealing with their infants.

### Limitations

One of the limitations of this study was the small sample size, which limits generalizability. In addition, our results may have been influenced by “selective participation” in that this group of the fathers volunteered to be part of the study, and their enthusiasm may have been an indication that they were the kind of fathers who wished to be more involved in their infants' lives. As Costigan and Cox (46) noted, fathers who participate in research tend to be more involved in family life than non-participating fathers.

Another limiting factor relates to a possible social desirability bias which may have played a role in the fathers' responses. Although the focus groups were led by an outside facilitator who conveyed to the fathers that she had very little to do with the study, the fathers nevertheless may have felt a need to paint their Baby Theater experience in a positive light. During the course of the study, they may also have sensed, on some level, a hope from the authors that this project would show positive outcomes. The fact that the performance was being videotaped may have also contributed to the fathers putting a positive spin on their experience. The fathers may also have felt that they needed to present themselves well in front of the other fathers in the group.

### Recommendations

Knowing that uninvolved fathers may not be drawn easily into partaking in a father-infant study, thought has to be given as to how best to recruit these fathers. We predict that the use of Baby Theater or other performing arts might be beneficial because, gradually, through word of mouth in various communities, it may become seen as an enjoyable, positive experience. The theater is currently funded with private and public sponsorship as well as ticket sales. For multiple Baby Theater productions to become viable, further funding would have to be obtained, which would cover the costs of the theater production as well as the traveling costs to various communities. Because Baby Theater has its own traveling stage in the form of the tent, and the props are easy to transport, Baby Theater could easily be performed outside its current theater venue directly within communities. This would eliminate the necessity of expensive travel into the city. Via word of mouth, fathers may become curious about what Baby Theater may offer.

Research is also needed to assess the lasting effects of the Baby Theater experience regarding the fathers' interest in, and involvement with, their children.

## Conclusion

This study assessed the responses of a small sample of South African fathers and their infants using the interpretative phenomenological approach. Their positive reactions to their experience indicated that father-infant Baby Theater may be a useful way to enable fathers to a have positive experience with their babies, enabling them to feel closer to their infants.

A non-clinical setting, such as a theater or show, in which fathers can be with their babies, might be an accessible as well as an acceptable point of entry to obtain information about what their babies enjoy. This will thereby create opportunities for joyful interaction and help to increase the fathers' involvement in their babies' lives. Further research with a larger sample and looking at the long term effects of the Baby Theater experience is needed.

## Data Availability Statement

The original contributions presented in the study are included in the article/supplementary material, further inquiries can be directed to the corresponding author/s.

## Ethics Statement

The studies involving human participants were reviewed and approved by Health Research Ethics Committee (HREC) of Stellenbosch University (Ref.# S17/10/266). Written informed consent to participate in this study was provided by the participants' legal guardian/next of kin.

## Author Contributions

BC: Main researcher, primary investigator, involved in the protocol, ethical submission, and analysis of data. AB: Supervisor, contributed to conceptualization of the study, protocol, and review of manuscript. AL: Co-Supervisor, contributed to protocol development, ethical review, and manuscript review. EW: Qualitative research integrity review and manuscript analysis. All authors contributed to the article and approved the submitted version.

## Conflict of Interest

The authors declare that the research was conducted in the absence of any commercial or financial relationships that could be construed as a potential conflict of interest.

## Footnotes

^1^As per South African Census racial classification. “Colored” referring to those of mixed-race descent.

^2^https://www.youtube.com/watch?v=_-vQ5GV55DU&feature=youtu.be&fbclid=IwAR1o0E70dObXSPdPbfHZSRp5Dm41oqTCkNtoo3PtLL-CuT8YTlt6PutJgwQ&ab_channel=MagnetTheatreZA (Accessed October 23, 2020).

^3^Afrikaans slang for “irritated”.
